# Experimental and Numerical Investigation of the Mechanical Properties of a CFRP Tendon–Wedge Assembly Loaded under Transverse Compressive Loading

**DOI:** 10.3390/ma16093305

**Published:** 2023-04-23

**Authors:** Qinghua Han, Xiwen Zheng, Lichen Wang

**Affiliations:** 1 School of Civil Engineering, Tianjin University, Tianjin 300072, China; qhhan@tju.edu.cn (Q.H.); zhengxiwen627@126.com (X.Z.); 2 Key Laboratory of Coast Civil Structure Safety of China Ministry of Education, Tianjin University, Tianjin 300072, China

**Keywords:** CFRP tendon–wedge assembly, transverse mechanical properties, size effect, stress state, integrated-wedge design

## Abstract

Carbon-fiber-reinforced polymer (CFRP) tendons have become a viable alternative to steel cables in cable roof structures owing to their high tensile strength, low weight, and resistance to corrosion. However, the effective anchoring of CFRP tendons is a challenge because of their poor transverse mechanical properties. Therefore, the mechanical properties of CFRP tendons and a tendon–wedge assembly under transverse compression were investigated by simulating the force environment of the CFRP tendon inside an integrated-wedge anchorage. The deformation of and local damage to CFRP tendons under transverse compression were explored using load–strain curves and full-field strain measured using digital image correlation. The experimental and numerical results show that large-diameter CFRP tendons with a length in the range of 90–110 mm had better cross-sectional deformation resistance and more stable transverse mechanical properties. Longer CFRP tendons with larger diameters have lower contact compressive stress and local maximal shear stress under the same transverse compressive load. Based on the analysis of the experimental and numerical results, we propose design suggestions for tendon size selection and integrated-wedge design details, such as the manufacturing materials of the wedge, the radius through the gap of the wedge, and the radial difference of the groove, to improve the anchoring properties and efficiency of the integrated-wedge anchorage.

## 1. Introduction

In the last few decades, steel cables have been successfully and widely used in cable structures. However, as the span of cable structures increases and the service environment becomes more complex, increasing amounts of corrosion and fatigue damage are being discovered on the steel cables of cable structures [1]. To solve these problems, several engineers have attempted to find new high-performance materials for replacing steel cables.

As an advanced nonmetallic material, carbon-fiber-reinforced polymer (CFRP) has been considered the ideal material for replacing steel cables since 1982 [2]. Regarding their lifetime and mechanical properties, carbon-fiber composites have the highest potential for replacing steel cables, compared to aramid, glass, or basalt, because of their high strength, light weight, no corrosion risk, excellent creep and fatigue resistance, low linear expansion coefficient, and relaxation [3,4,5,6]. Existing studies have indicated that the application of CFRP could effectively solve the problems of corrosion, fatigue, self-weight, and prestress loss in the application of steel cables and could improve the overall performance of cable structures [7,8,9]. However, CFRP tendons are typically brittle orthotropic materials with weak transverse mechanical properties that pose a great challenge to anchoring CFRP tendons [10]. Therefore, the development of a practical and cost-effective anchorage system that could safely anchor CFRP tendons to the level of their ultimate tensile strength, without premature fractures due to transverse stresses from the anchorage, is a key issue for the application of CFRP cables in cable structures.

Extensive research has been conducted on mechanical-type anchorages that are suitable for cable roof structures owing to their small size, light weight, and easy installation [11]. In 1998, Sayed-Ahmed and Shrive [12] developed a type of separated-wedge (4-piece) anchorage that comprised three design concepts: small-angle wedge (no greater than 3°), soft-metal sleeve, and angle differences. Static tensile tests showed that CFRP tendons with a diameter of 8 mm could be anchored to 2300 MPa. Al-Mayah et al. [13] proposed the curved-angle concept to achieve uniform contact pressure along the CFRP. In tensile tests, an anchorage with a radius of 1900 mm achieved the best performance, yielding an average failure load of 152 kN (2190 MPa). Heydarinouri et al. [14] applied a 3-separate-aluminum-wedge anchorage to anchor 8 mm CFRP tendons to 2371 MPa. In terms of anchoring failure strength, this type of separated-wedge anchorage has not achieved a highly efficient anchoring performance. Schmidt et al. [15] proposed the novel concept of an integrated wedge that solved the problem of coordinating the work of the separated wedges and reduced the number of components in the anchorage, thus improving the stability of the anchoring performance and facilitating installation. The anchorage had an outward angle of 3.4° and an angular difference of 0.4°. A static tensile test showed that this type of integrated-wedge anchorage could anchor CFRP tendons with an 8 mm diameter to 2900 MPa with very high anchoring efficiency. A three-dimensional model of an integrated-wedge anchorage was built on the basis of a local coordinate system [16]. This finite-element model accurately simulated circumferential barrel strains. On the basis of this exciting development in integrated-wedge anchorages, Schmidt et al. [17] proposed a novel ductile anchorage system by adding a ductile mechanism as part of the anchorage system. The tensile capacity of the final modified anchorage reached a mean value of 2986 MPa, enabling a change in the failure mode from brittle to ductile. Although the integrated-wedge anchorage exhibited excellent anchoring properties, the lack of a standardized design method has hindered its widespread application owing to the unclear transverse mechanical properties of CFRP tendons inside a wedge. The failure of CFRP tendons in practical applications cannot be described based on the strength of the uniaxial stress–strain behavior obtained from standard material-property tests [18]. In the case of multidirectional loading, the failure of a CFRP tendon is especially considered to be strain-controlled. Moreover, the overall performance of a CFRP tendon anchorage system obtained from an anchoring tensile test is not representative of the local stress state and damage [19]. Therefore, a deeper understanding of the transverse mechanical properties of CFRP tendons inside an actual force environment is critical for the optimal design of an anchorage. Terrasi et al. [20] performed a compression test on a CFRP tendon to determine the contact stiffness between the CFRP tendon and wedge in order to simulate the anchorage system as accurately as possible. Han et al. [21] investigated the effect of the diameter and length on the transverse compressive mechanical properties of CFRP tendons by applying transverse line loads. Tanks et al. [22] studied the effect of the surface finish on the transverse compressive mechanical properties of CFRP tendons with a 12.7 mm diameter. The test results showed that tendons with a smooth round surface exhibited the best transverse compressive deformation and load-carrying capacity. A 2D simulation method was proposed for the early stages of designing an anchorage prototype. Fang et al. [23] conducted transverse static and low-velocity impact tests on prestressed CFRP tendons and carbon strands. The transverse compressive load capacity of CFRP tendons decreased by 28% under low-velocity impact loading compared to those with a static load. In summary, although there are some studies on the transverse compressive properties of CFRP tendons, the studies regarding the effect of the tendon size on transverse mechanical properties, the mechanical response of the CFRP tendon–wedge assembly, and the stress state of the tendon are not sufficient, which hinders attempts to further refine the optimization of anchoring performance for integrated-wedge anchorages.

In this paper, the transverse compressive mechanical properties of a CFRP tendon–wedge assembly are investigated using experimental and numerical methods. The experimental part focuses on the transverse mechanical properties of and damage to CFRP tendons. A tendon with a relatively large diameter and a length of 90–110 mm has stable transverse mechanical properties. Excellent grip was obtained for the tendons when the contact area between the tendons and aluminum plates was greater than 92%. The simulation part focuses on an analysis of the stress state of a CFRP tendon under a transverse load. A design value for the gap of the integrated wedge is proposed for different sizes of CFRP tendons. The main research objective is to propose some design suggestions and optimized design values for tendon-size selection and integrated-wedge design details, such as the manufacturing material of the wedge, the radius through the gap of the wedge, the outside angle of the wedge, and the radial difference of the groove. These results will be very useful for understanding the transverse compressive properties of CFRP tendon–wedge assemblies and promoting the performance optimization of integrated wedges.

## 2. Materials and Methods

### 2.1. Materials and Test Specimens

**Figure 1:**
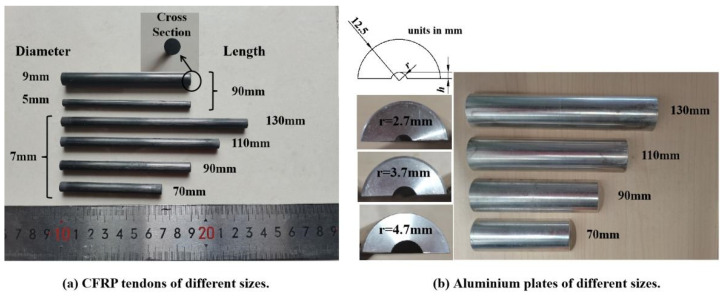
Specimens of different sizes.

CFRP tendon specimens with smooth surfaces were composed of polyacrylonitrile-based carbon fibers T700-12K (Weihai Guangwei Composite Material Co., Weihai, China) impregnated with polyfunctional epoxy resin CP-02A/B (Hangmo New Materials Group Co. Ltd., Huzhou, China) via a pultrusion process. The volumetric fraction of the carbon fiber in the CFRP tendons was designed to be 65%. As shown in Figure 1a, 3 diameters, of 5, 7, and 9 mm, were tested for CFRP tendon specimens having a length of 90 mm. Additionally, 3 sample lengths, of 70, 110, and 130 mm, were selected for the tendon specimens with a diameter of 7 mm, according to the commonly used size specifications of existing anchorages [10,24]. The end sections of the CFRP tendons were cut flat using digital image correlation (DIC).

The two parts of the upper and lower semicircular aluminum plates (A6061-T6, yield strength of 230 MPa) with grooves were manufactured to simulate a wedge. To avoid loading failure caused by contact between the upper and lower plates during the loading process, a sufficient gap should be left between the plates. As shown in Table 1 and Figure 1b, the gaps between the aluminum plates were 1.4, 1.8, and 2.2 mm for CFRP tendon specimens of 5, 7, and 9 mm, respectively. The length of the aluminum plate was the same as that of the CFRP tendon specimen, as shown in Figure 1b. In transverse compression testing, three influencing factors, tendon diameter, length, and aluminum plate, were considered. The geometric details and the number of CFRP tendons and aluminum plate specimens are listed in Table 1.

### 2.2. Test Method

**Figure 2:**
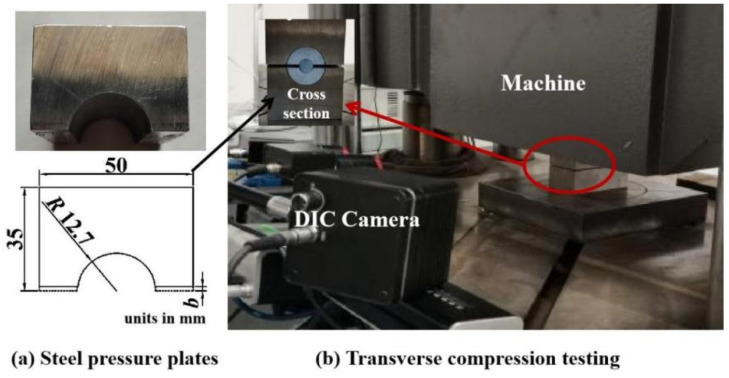
Testing fixtures and setup.

As shown in Figure 2a, a pair of steel plates with cross-sectional dimensions of 35 mm × 50 mm were used to transfer the load from the testing machine to the aluminum plate and CFRP tendon specimens. The material of the steel plate was 42CrMo (after annealing) with a yield strength of 930 MPa and a hardness of 50–60 HRC to ensure sufficient rigidity. A groove that matched the outer diameter of the semicircular aluminum plate was created in the steel plate. As shown in Figure 2b, in transverse compression testing, the CFRP tendon, aluminum plate, and steel plate were assembled on a servo-hydraulic testing machine (1000 kN capacity) and loaded to failure at a loading rate of 1.5 mm/min [25]. The vertical strain of the CFRP tendon end-face and the full-field strain of the end-face of the CFRP tendon and aluminum plates were measured using DIC during the entire loading process. The gauge length of the vertical strain was defined as the diameter of the tendon end-face parallel to the applied loading direction. The end-face of the CFRP tendon and aluminum plate were sprayed spot to cooperate with the DIC technique.

## 3. Experimental Results and Discussion

### 3.1. Deformation and Damage Mode of CFRP Tendons

**Figure 3:**
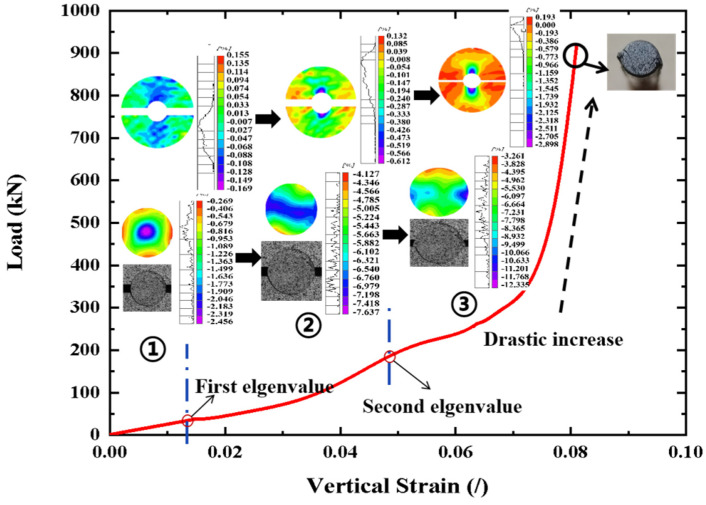
Typical load–strain curve of CFRP tendon under transverse load.

The typical load–strain curve (triplicate-average results) of the CFRP tendon (D2) in transverse compression is shown in Figure 3. The curve can be divided into three stages according to the characteristics of the strain distribution on the end-face of the tendon.

In the first stage, the maximum compressive strain of the CFRP tendon end-face was concentrated in the middle region of the end-face and formed a decreasing pattern along the radial direction, which presented a force transfer pattern, larger in the middle and smaller on both sides. The image in Figure 3 shows that the end-face of the tendon remained round at this stage. As the transverse load increased, the deformation of the tendon exhibited linear elasticity owing to the linear feature of the load–strain curve. Additionally, the strain plots of the aluminum plate end-face showed the characteristics of middle pressure and outwards warping of the two sides. However, the boundary between the pulling area and the pressure area was not obvious. The tendon and aluminum plate were in a good contact state.

In the second stage, the maximum compressive strain on the tendon end-face expanded gradually from the middle region to both sides and eventually formed a laminar distribution pattern from small to large to small along the vertical direction. This strain distribution indicated that the forces were transferred efficiently at the tendon cross-section. The geometry of the tendon end-face changed from circular to elliptical, indicating that nonuniform deformation occurred in the tendon as the transverse load continued to increase. Accordingly, the load–strain curve began to exhibit nonlinear characteristics in the second stage. For the aluminum plate, the maximum compressive strain was located in the middle region in contact with the tendon, and the strain magnitude difference was small. A majority of the region on the aluminum plate end-face was in a pressure state, and the phenomenon of outwards warping on the two sides almost disappeared. Based on the full-field strain results of the tendon and aluminum plate, it was observed that the contact area between the tendon and aluminum plate was larger than that in the first stage owing to the nonuniform deformation of the tendon. Therefore, a better-fitting contact promoted the stability of the load transfer. For a quantitative comparison, the dividing point (load value) between the first and second stages of the load–strain curve was defined as the first eigenvalue, as shown in Figure 3.

In the third stage, the maximum compressive strain was concentrated on both sides of the tendon end-face and the remaining region of the strain distribution on the end-face was approximately uniform (large green area in the strain plots). The uniform strain distribution implies that the cross-section of the tendon can transmit force quickly and efficiently with only a small deformation. Correspondingly, a drastic increase occurs in the load–strain curve as the load increases, which reflects the force transmission characteristics of the tendon cross-section at this stage. The photograph of the tendon end-face shows that the eccentricity of the elliptical geometry of the tendon end-face increases, which is beneficial for a better fit between the tendon and the groove of the aluminum plate. Two rib-like protrusions (diametrically opposite) developed along the length of the tendon as the load increased. The rib-like protrusions were produced because some material from the tendon was forced into the gap between the aluminum plates as the gap was reduced under an increasing transverse load. The edge of the groove of the aluminum plate exhibited shearing and extrusion on the rib-like protrusions of the tendon, which caused the appearance of maximum compressive strain concentration on both sides of the tendon. It is clear from the images that some visible cracks located near the gap between the aluminum plates oriented at 40°–60° from the loading direction occurred on the tendon end-face. Based on the results of the strain distribution, deformation, and damage, the aluminum plate had an excellent grip on the tendon, which was able to withstand large transverse loads with small deformations in the third stage. When the load was stopped owing to the contact between the aluminum plates or the load limitation of the machine, the CFRP tendon remained intact and could continue to carry the load. The rib-like protrusions of the tendon were flattened by the aluminum plates and sheared off in some cases. The dividing point (load value) between the second and third stages of the load–strain curve was defined as the second eigenvalue, as shown in Figure 3.

It is worth noting that a sharply rising section appears on the load–strain curve in the third stage. This is beneficial for improving anchoring efficiency. This is because the aluminum plate grooves have a good circumferential constraint on the CFRP tendons under a sufficiently large contact area, resulting in the aluminum plates having an excellent grip on the tendon. Therefore, ensuring a sufficiently large contact area between the tendon and aluminum plates is critical for producing an excellent grip. Based on the DIC results and images, the area ratio of the contact area between the tendon and grooves of the aluminum plates to the surface area of the tendon was measured and calculated to be between 91% and 93% for all the specimens when a sharp increase occurred in the load–strain curve in the third stage, as shown in Table 2. Thus, an area ratio of at least 92% was suggested to ensure a sufficient contact area.

### 3.2. Effect of Diameter on the Compressive Properties of CFRP Tendons

**Figure 4:**
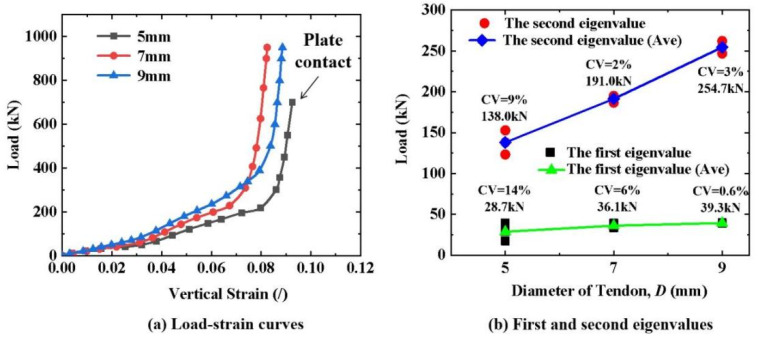
Transverse compressive properties of CFRP tendons with different diameters.

As shown in Figure 4a, the load–strain curves (triplicate-average results) of the CFRP tendons with diameters of 5, 7, and 9 mm exhibited the same trend. The load–strain curves almost overlapped at the beginning. As the load increased, the slope of the load–strain curve increased for the larger-diameter CFRP tendons. This means that the cross-section of the tendons with a larger diameter had better resistance to deformation under the same transverse load. Additionally, the CFRP tendons with different diameters exhibited the same deformation and damage mode characteristics under a transverse load. Based on the three-stage division method of the load–strain curve, the first and second eigenvalues were recorded for all the specimens, as shown in Figure 4b. The first and second eigenvalues increased approximately linearly as the diameter increased. This also demonstrates that the cross-section of tendons with a larger diameter had better resistance to deformation. Because the crack occurred in the third stage, local material damage is more likely to occur in tendons with smaller diameters under the same transverse load. For the first and second eigenvalues, the coefficient of variation (CV) of the 5 mm diameter specimens was significantly larger than that of the 7 and 9 mm diameter specimens. In summary, the large-diameter CFRP tendons exhibited better cross-sectional deformation resistance and more stable transverse mechanical properties.

### 3.3. Effect of Length on the Compressive Properties of CFRP Tendons

**Figure 5:**
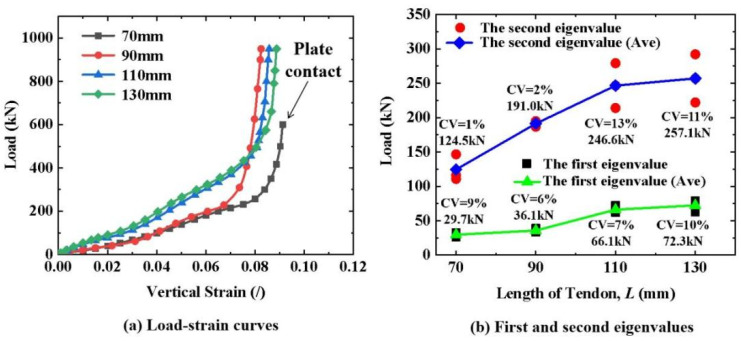
Transverse compressive properties of CFRP tendons with different lengths.

As shown in Figure 5a, the load–strain curves (triplicate-average results) of the 7 mm diameter CFRP tendons with different lengths exhibited the same trend. The load–strain curves for the 110 mm- and 130 mm-long tendons almost coincided. Moreover, they had a larger slope compared to those of the 70 mm- and 90 mm-long tendons. Therefore, the cross-section of the tendons with 110 mm and 130 mm lengths had better resistance to deformation under the same transverse load. This resistance to deformation did not increase when the length was greater than 110 mm. Although the first and second stages of the load–strain curve for the 90 mm-long tendon coincided with that of the 70 mm-long tendon, the sharply rising section of the load–strain curve had the same characteristics as those of the 110 mm- and 130 mm-long tendons. This means that the aluminum plates also had an excellent grip on the 90 mm-long tendon. The CFRP tendons of different lengths had the same deformation and damage mode characteristics under a transverse load. The statistical results of the first and second eigenvalues showed characteristics consistent with the load–strain curves. For the first eigenvalue, those of the 110 mm- and 130 mm-long tendons were significantly higher than those of the 70 mm- and 90 mm-long tendons. The second eigenvalue increased approximately linearly as the length increased, until the length was greater than 110 mm. The 90 mm-long tendons had more stable transverse mechanical properties than the tendons of other lengths owing to their having the smallest CV of the first and second eigenvalues. Considering these two factors of cross-sectional deformation resistance and mechanical stability, it is recommended to use 90–110 mm-long CFRP tendons.

### 3.4. Effect of Aluminum Plates on the Compressive Properties of CFRP Tendons

**Figure 6:**
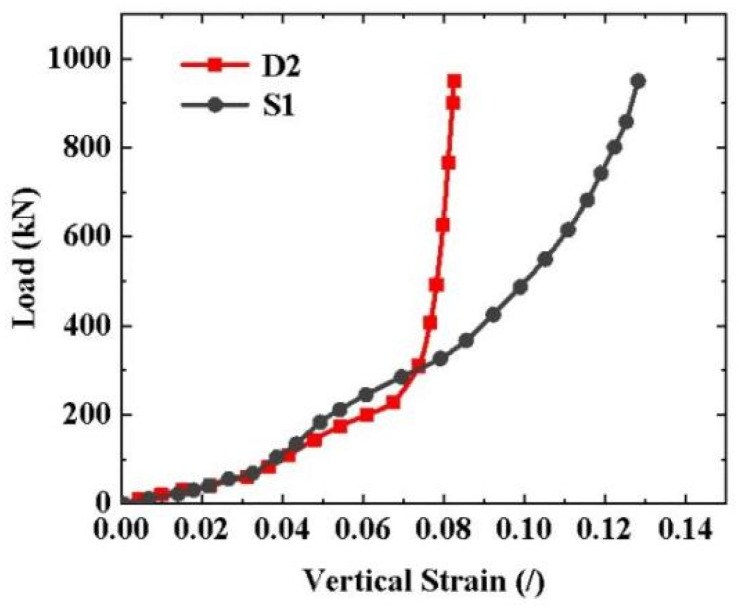
Typical load–strain curves of the CFRP tendons with and without the aluminum plate.

As shown in Figure 6, the two load–strain curves almost overlapped in the first and second stages but had an obvious difference in the third stage. The sharply rising phenomenon of the load–strain curve occurred in the case with aluminum plates, but not in the case without aluminum plates. This means that the plates made of hard steel did not have a good grip on the CFRP tendon. This is because aluminum plates, which have a low elastic modulus, can increase the contact area with the tendon through plastic deformation. Therefore, the presence of a soft metal is critical for creating a good grip on the CFRP tendons and to improve the anchoring efficiency. The experimental results show that aluminum had a good grip on the CFRP tendon and is the recommended material for manufacturing the wedge.

## 4. Simulation Analysis Results and Discussion

### 4.1. Finite Element Model (FEM)

**Figure 7:**
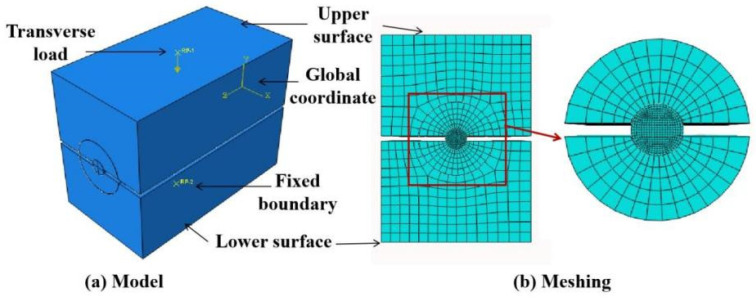
FEM of transverse compression testing.

A three-dimensional FEM was built using the commercial software Abaqus 2020 to simulate transverse compression testing. As shown in Figure 7a, the coordinate system was established with the axial direction of the CFRP tendon along the Z direction and the radial direction in the X and Y directions. The CFRP was set as an orthotropic material [26,27], and the aluminum and steel plates were assumed to be linearly elastic materials, as shown in Table 3. To simulate the contact behavior of the CFRP tendon–aluminum plate and the aluminum plate–steel pressure plate, surface-to-surface contact with limited sliding and a specific friction coefficient of 0.24 [28,29] were set. An eight-node linear brick with reduced integration and an hourglass-control (C3D8R) element were used for the CFRP. The model was fixed by applying a Y-direction constraint to the lower surface of the steel pressure plate. The transverse compression load was set as the reference point of the upper steel pressure plate. As shown in Figure 7b, the meshing size of the cross-section of the steel pressure plate and the CFRP tendons were 3 mm and 0.5 mm, respectively. The meshing size gradually transitioned from 3 mm to 0.5 mm from the outer surface to the inner surface for the cross-section of the aluminum plate.

### 4.2. Comparison between FEM and Experimental Results

**Figure 8:**
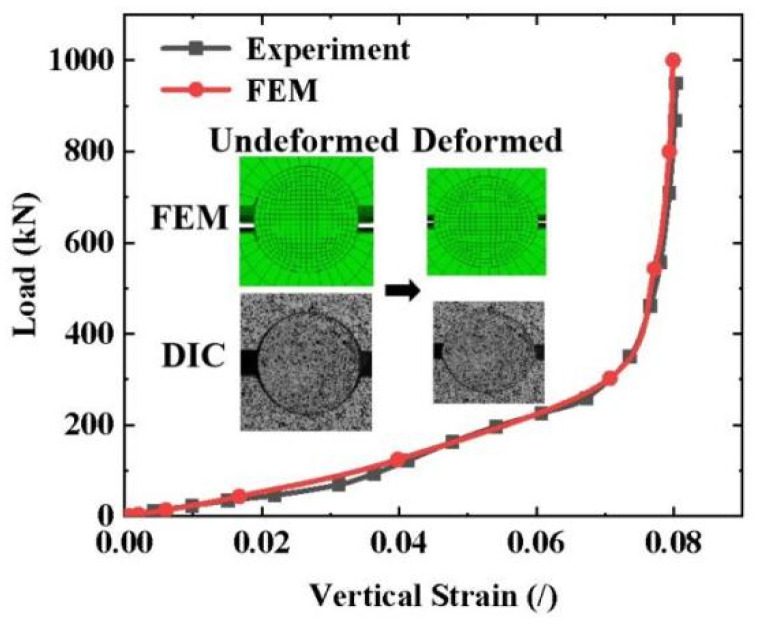
Comparison of the load–strain curve and deformation between the FEM and the experimental results.

The FEM and experimental results were compared using the load–strain curves, end-face deformation, and end-face strain fields of the CFRP tendons. As shown in Figure 8, the FEM results of the load–strain curve of a typical specimen (CFRP tendons with 7 mm diameter and 90 mm length) were in good agreement with the experimental results in terms of the trend and values. Compared to the experimental results, the curves obtained from the FEM were smoother owing to the absence of dimensional machining errors in the FEM. As the load increased, the shape of the end-face in the CFRP tendon in the FEM changed from circular to elliptical, which was consistent with the deformation law obtained from the test.

**Figure 9:**
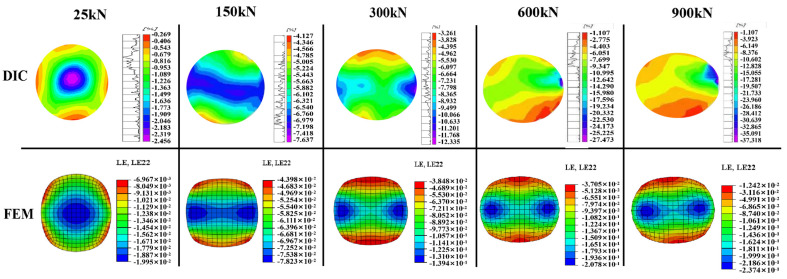
Comparison of full−field strain under different loading stages in the FEM and DIC results.

A comparison of the FEM results with the full-field strain measured using DIC at different loading stages for a typical specimen is presented in Figure 9. The FEM and DIC results were in good agreement in terms of the strain distribution mode, strain magnitude, and deformation law. In the loading stage of 0–300 kN, there was only a small difference between the FEM and DIC results, including the strain magnitude. This was likely because of the nonuniformity of the microstructure of the CFRP tendon that was not considered in the FEM. As the load continued to increase, the discrepancy between the FEM and DIC results increased slightly. While maintaining a consistent overall trend of strain distribution, the discrepancy in the maximum strain increased to approximately 30%, and some local strain variations could not be represented in the FEM results. The increased discrepancy was caused by the local crack damage in the tendon, particularly by the appearance of rib-like protrusions. This local damage was not effectively simulated by the FEM. For each tendon specimen, the strain fields obtained using DIC had slight local differences owing to slight differences in the local damage and irregular deformation. Nevertheless, the overall trend of the strain distribution was consistent. Therefore, the increased discrepancy between the FEM and DIC results is expected to be associated with the variability caused by the damage. In conclusion, the FEM can accurately reflect the transverse mechanical response of the CFRP tendon in transverse compression testing and can be used for numerical simulation analysis.

### 4.3. Contact Compressive Stress between CFRP Tendon and Aluminum Plate

**Figure 10:**
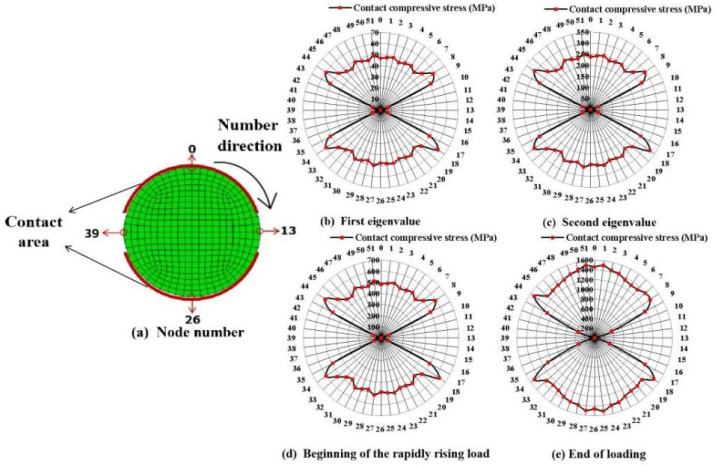
Distribution and magnitude of contact compressive stress on the contact surface under different loads.

The contact compressive stress distribution and the magnitude of the contact surface between the tendon and aluminum plate groove under different loads, including the first eigenvalue (36 kN), second eigenvalue (191 kN), beginning of the rapidly rising load (353 kN), and the end of loading (1000 kN), were investigated for a typical specimen (CFRP tendons with a 7 mm diameter and 90 mm length) through the FEM. As shown in Figure 10a, 52 nodes were numbered on the cross-section of the tendon FEM in a clockwise direction according to the mesh size. The contact compressive stress of each node was extracted to plot the envelope, as shown in Figure 10b–e. The distribution pattern of the contact compressive stress along the circumference of the cross-section was approximately symmetrical and nonuniform. This symmetry weakened as the load increased because of the large and uneven deformation of the tendon and aluminum plates under a large load. In terms of the magnitude of the contact compressive stress, the stress mutation (nodes 8, 18, 34, and 44) and noncontact status (contact stress is 0) appeared near the gap between the aluminum plates, which was caused by the change in the boundary conditions of the tendon at the edge of the aluminum plate groove. By removing the contact stress mutation and 0 values, the average contact compressive stress of the other nodes were 48 MPa, 248 MPa, 490 MPa, and 1378 MPa, respectively, with a CV of approximately 5% under the four typical loads. The corresponding stress mutation values were 63 MPa, 324 MPa, 634 MPa, and 1510 MPa, with discrepancies of 31%, 31%, 29%, and 10%, respectively, compared to their average. Therefore, the degree of contact compressive stress mutation decreased significantly as the load increased. This was because a larger contact area and more stable contact action were obtained through the deformation of the tendon and aluminum plate. When the load increased to the second eigenvalue, no damage occurred to the tendon, and the contact compressive stress on the surface reached approximately 250 MPa, which was already greater than the compressive strength of 125 MPa measured via the standard test method [29]. When the loading stopped, the tendon was able to withstand the transverse load with a very high contact compressive stress of approximately 1500 MPa. The dramatic increase in the load-carrying capacity of the CFRP tendon was caused by the effective circumferential constraint provided by the aluminum plate grooves. Therefore, considering the actual force environment is critical for evaluating the transverse carrying capacity of CFRP tendons in anchorages.

### 4.4. Shear Stress of CFRP Tendon

**Figure 11:**
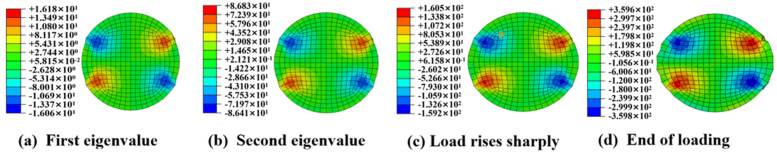
Shear stress distribution of the CFRP tendon cross−section under different loads.

The distribution of the shear stress on the cross-section of a typical specimen (CFRP tendons with a 7 mm diameter and 90 mm length) under four typical loads is shown in Figure 11. The shear stress distribution pattern was symmetrical and remained constant until the loading stopped. The maximum shear stress was located adjacent to the region of the contact compressive stress mutation and oriented 50°–60° from the transverse loading direction, which was almost identical to the direction of the cracks on the tendon end-face. Therefore, the contact compressive stress mutation was likely the reason for the generation of the large local shear stress. Additionally, the maximum shear stress reached 360 MPa (Figure 11d) at the end of the loading, which was greater than the transverse shear strength of 320 MPa of the CFRP tendons [30]. It can be inferred that the crack damage on the tendon end-face was caused by excessive shear stress. The transverse shear strength of the CFRP tendons based on the actual force conditions can potentially be used as the failure criterion for designing an anchorage.

### 4.5. Effect of Size on Stress State of CFRP Tendon

**Figure 12:**
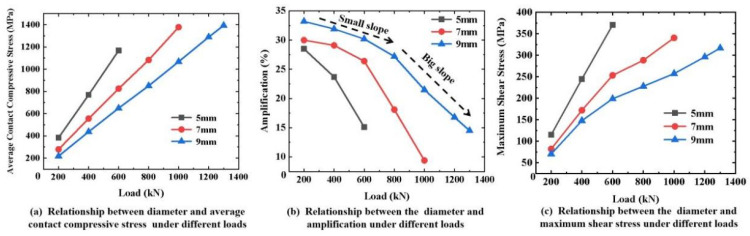
Effect of diameter on the stress state of CFRP tendons.

FEMs with different diameters and lengths were built to investigate the effect of size on the contact compressive stress and shear stress of the CFRP tendons. As shown in Figure 12a, the average contact compressive stress decreased with an increasing diameter under the same transverse load. As shown in Figure 12b, the increase in the amplitude between the average contact compressive stress and mutation decreased as the diameter decreased and the load increased. In terms of the contact compressive stress, a higher contact compressive stress appeared when using smaller diameter tendons under the same transverse load. However, a more uniform stress distribution can be obtained. As shown in Figure 12c, the maximum shear stress decreased as the diameter increased. The generation of shear stress requires the consideration of two influencing factors, the mutation degree and the contact compressive stress absolute value. As the diameter increased, the change law in the magnitude and mutation amplitude of the contact compressive stress were opposite. The magnitude of the contact compressive stress is a determinant of the maximum shear stress compared to the mutation. Therefore, a CFRP tendon with a larger diameter has a lower local maximum shear stress.

**Figure 13:**
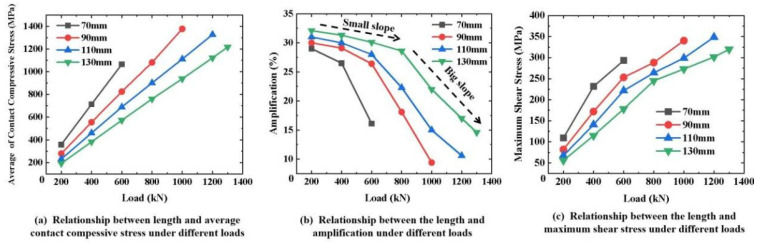
Effect of length on the stress state of CFRP tendons.

The effect of length on the stress state of the CFRP tendons was essentially the same as that of the diameter, as shown in Figure 13. In summary, the contact compressive stress mutation amplitude increased on the surface of the CFRP tendons with larger diameters and longer lengths, but the contact compressive stress and the local maximum shear stress were lower. Therefore, to satisfy the design value of the tensile load capacity of CFRP tendons, the selection of CFRP tendons with a large diameter and length can reduce the contact compressive stress, thus avoiding the local damage to CFRP tendons caused by excessive shear stress in the design of anchorages.

### 4.6. Effect of Radius Difference on the Stress State of CFRP Tendons

**Figure 14:**
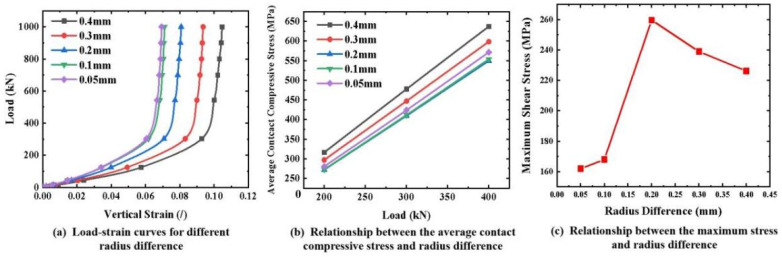
Effect of radius difference on the stress state of CFRP tendons.

In practice, anchorage components are designed with a certain dimensional difference to facilitate assembly. As shown in Figure 1b, a radius difference of 0.2 mm between the CFRP tendon and the groove of the aluminum plate was designed for transverse compression testing. A large range of parametric analyses on the effect of the radius difference on the stress state of the CFRP tendon were conducted using an FEM. As shown in Figure 14a, when the radius difference was smaller, the vertical strain of the tendon was smaller under the same transverse load. This reduction effect was not obvious until the radius difference was less than 0.1 mm. In terms of the contact compressive stress, the average contact compressive stress decreased with a decreasing radius difference until the radius difference was 0.1 mm, as shown in Figure 14b. With respect to the shear stress, when the radius difference was less than 0.1 mm, the shear stress on the cross-section of the CFRP tendon was in a smaller state, as shown in Figure 14c. In summary, a radius difference of 0.1 mm is suggested in the design of an integrated wedge, which is beneficial for reducing the compressive stress and shear stress of the CFRP tendon.

### 4.7. Required Minimum Wedge Gap

**Figure 15:**
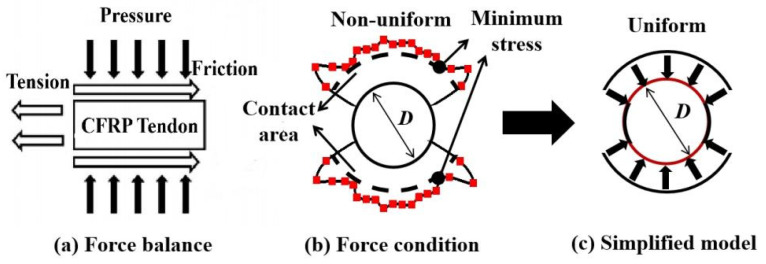
Simplified method of force conditions for CFRP tendons.

For the integrated-wedge anchorage, the gripping force on the CFRP tendon was generated by the gradual closing of the wedge gap as the wedge continued to enter the barrel. However, the gripping force on the tendon disappeared when the gap was closed completely, and the tendons slipped rapidly under high tension [15]. Therefore, a suitable gap design value is critical for ensuring a sufficient gripping force generated by the wedge. Based on the force balance law between the tension and friction of the CFRP tendon, as shown in Figure 15a, the required minimum gap was analyzed using the FEM of transverse compression testing. On the one hand, the nonuniform distribution of the contact compressive stress on the surface of the CFRP tendon obtained from the FEM was simplified to a uniform distribution by reducing all contact compressive stress to the minimum of them, as shown in Figure 15b,c. In contrast, the contact compressive stress distribution was set as uniform along the length of the CFRP tendon. Although the simplified calculation method does not reflect the nonuniform stress status on the tendon surface inside the wedge, the longitudinal tensile stress of the tendon calculated using this method is conservatively low and can be used as the control value for the minimum gap design. The longitudinal tensile stress of the CFRP tendons can be calculated using Equation (1):

(1) $$\sigma =\frac{\mu P}{A}$$

where *σ* is the longitudinal tensile stress of the CFRP tendon, *P* is the contact force equal to the minimum contact compressive stress (FEM result) multiplied by the contact area (FEM result), *μ* is the friction coefficient (0.24) [26,27] between the CFRP tendon and the aluminum plate, and *A* is the cross-sectional area of the CFRP tendon.

**Figure 16:**
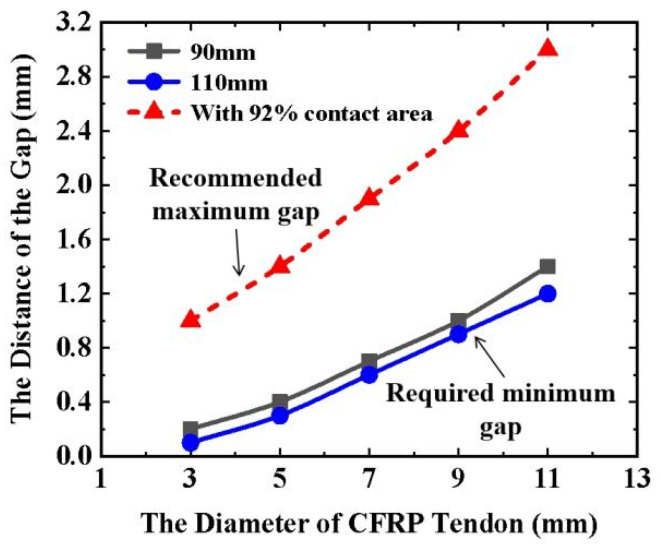
Relationship between the diameter and required minimum/recommended maximum gap.

Using 3100 MPa as the target tensile strength of the CFRP tendon [15], the required minimum gap values were investigated using the FEM and Equation (1). For example, a transverse compression model with a particular gap was built in the FEM for a CFRP tendon. The transverse load was conducted on the FEM until the gap completely closed. Then, the minimum contact compressive stress and contact area were extracted from the FEM results to calculate the longitudinal tensile stress using Equation (1). When the longitudinal tensile stress achieves 3100 MPa (a theoretical design value for the strength of the CFRP), the corresponding gap is the required minimum gap for the size of the CFRP tendon. The lengths of 90 and 110 mm were selected in the analytical model for the CFRP tendon owing to their good transverse mechanical properties (as mentioned in Section 3.3). The statistical results for the CFRP tendons with different diameters are shown in Figure 16. Compared to the 90 mm-long tendons, the 110 mm-long CFRP tendons with the same diameter required a larger gap to obtain the target tensile strength. As the diameter of the CFRP tendons increased, the required minimum gap increased. Equation (2) was obtained by fitting the relationship between the minimum gap required and the diameter of the CFRP tendons:

(2) $$g_{\min}=\left\{ \begin{array}{l} 7.14d^{2}\times 10^{-3}+0.05d\;(L=90\;\mathrm{mm})\\ 3.58d^{2}\times 10^{-3}+0.09d-0.214\;(L=110\;\mathrm{mm}) \end{array}\right.$$

where *g*_min_ denotes the required minimum gap, *d* denotes the diameter of the CFRP tendon, and *L* denotes the length of the CFRP tendon. As a result, the required minimum gap can be obtained for CFRP tendons with different diameters and lengths. As mentioned in Section 3.1, at least 92% of the area ratio is suggested to ensure a sufficient contact area for obtaining a good grip on the CFRP tendon. The recommended maximum gap can ensure a sufficient contact area greater than or equal to 92%. Therefore, the recommended maximum gaps calculated for different diameters of CFRP tendons at a contact area ratio of 92% are shown in Figure 16. Equation (3) was obtained by fitting the relationship between the recommended maximum gap and the diameter of the CFRP tendons:

(3) $$g_{\max}=7.14d^{2}\times 10^{-3}+0.15d+0.482$$

where *g*_max_ is the recommended maximum gap and *d* is the diameter of the CFRP tendon. This gap is referred to as the recommended maximum gap for designing an integrated wedge to ensure an adequate initial contact area between the CFRP tendon and the wedge.

### 4.8. Tendon Size Selection and Integrated-Wedge Design Suggestion

A good grip of the wedge on the CFRP tendon is critical for improving the anchoring efficiency of the anchorage. Based on the experimental and FEM analysis results, some suggestions for tendon size selection and integrated-wedge design details are proposed to obtain a good grip of the wedge on the CFRP tendon.

In terms of the CFRP tendon size, large-diameter CFRP tendons with lengths ranging from 90 to 110 mm have better cross-sectional deformation resistance and more stable transverse mechanical properties. The contact compressive stress mutation amplitude increases with a larger diameter and longer length, but the contact compressive stress and local maximum shear stress will be lower. To obtain stable transverse mechanical properties and avoid excessive local shear stress, a relatively large diameter of 90–110 mm is suggested for selecting CFRP tendons.

**Figure 17:**
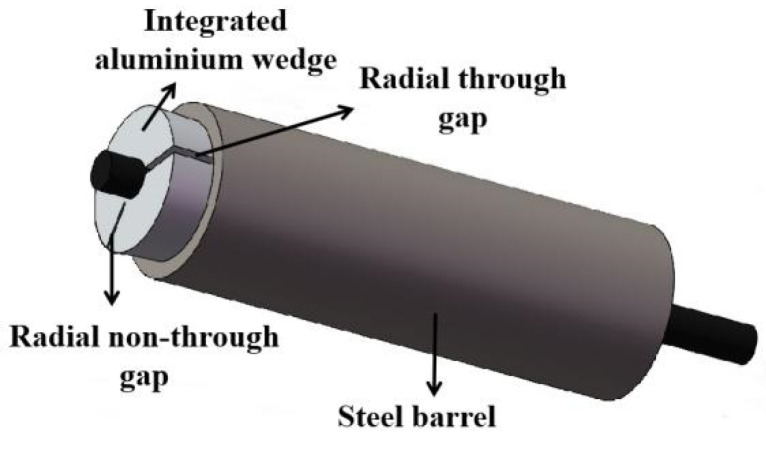
Integral wedge type anchorage.

The integrated wedge is the most important component of an integrated-wedge anchorage. As shown in Figure 17, the manufacturing material, radial through-gap of the wedge, outside angle of the wedge, and radius difference of the groove are some key design details of the integrated wedge. In this research, the outside angle of the wedge was not considered during transverse compression testing, as the transverse mechanical cross-section of the CFRP tendon was the focus of this research. Therefore, some design suggestions are proposed for choosing the material, gap, and radius differences. First, using soft metal can generate a good grip on the CFRP tendons. Hence, aluminum is suggested for manufacturing the wedge owing to its excellent grip on the CFRP tendon in the test. Second, the radial through-gap is critical to ensure the deformation capacity of the integrated wedge as it enters the barrel. The control range of the gap value is calculated using Equations (2) and (3), and the design value should be chosen within the larger side of this range. Third, a radius difference of 0.1 mm is proposed for the design of the radius of the wedge groove, which is beneficial for reducing the compressive and shear stresses of the CFRP tendon. Lastly, in the testing, the rib-like protrusions of the CFRP tendon were cut by the edge of the aluminum plate groove. Therefore, a rounding treatment is suggested for the edge of the integrated wedge that is in contact with the CFRP tendon to reduce the probability of local damage to the tendon.

## 5. Conclusions

A series of transverse compression tests were conducted on the CFRP tendon–wedge assembly, which is a commonly used anchorage method for CFRP cables. Based on the experimental results, a numerical analysis was performed using FEM. The following conclusions were drawn.

(1) The load–vertical strain curve of CFRP tendons under a transverse load can be divided into three typical stages according to the vertical strain distribution characteristics of the end-face. As the transverse load increased, the contact area between the CFRP tendon and aluminum plate grooves increased and the contact fitting became more stable. In the third stage, rib-like protrusions caused by the shearing and extrusion of the aluminum plate edges were generated along the length of the CFRP tendon. A sharply rising section appeared on the load–strain curve in the third stage, indicating that the aluminum plates had an excellent grip on the tendon. Ensuring a sufficient contact area of 92% between the tendons and aluminum plates is critical for producing an excellent grip. Towards the end of loading, some visible cracks appeared on the CFRP end-face owing to the excessive local shear stress.

(2) For satisfying the design value of the tensile load capacity of the CFRP cable, a relatively large diameter of 90–110 mm in length is suggested when selecting the CFRP tendons. This length is beneficial for obtaining stable transverse mechanical properties and for avoiding local damage to CFRP tendons due to excessive shear stress.

(3) Based on the experimental and FEM results and analyses, some design suggestions for tendon size selection and integrated-wedge design details are proposed to improve the anchoring properties and efficiency of the integrated-wedge anchorage. First, aluminum is the recommended material for manufacturing the wedge. Second, we suggest that the design value of the radial through-gap is selected within the larger side of the control range. Third, the optimization value of the radius difference should be 0.1 mm. Lastly, a rounding treatment is suggested for the edge of the integrated wedge.

## Figures and Tables

**Table 1:** Size and number of specimens.

No.	Number	CFRP Tendon		Aluminum Plate		
		*D* (mm)	*L* (mm)	*R* (mm)	*r* (mm)	*h* (mm)
D1	3	5	90	12.5	2.7	1.8
D2	3	7	90	12.5	3.7	2.6
D3	3	9	90	12.5	4.7	3.4
L1	3	7	70	12.5	3.7	2.6
L2	3	7	110	12.5	3.7	2.6
L3	3	7	130	12.5	3.7	2.6
S1	3	7	90	-	-	-

Note: *D* denotes diameter of CFRP tendons; *L* denotes length of CFRP tendons; *R* denotes outer radius of aluminum plate; *r* denotes inner radius of aluminum plate; *h* denotes actual depth of groove in aluminum plate.

**Table 2:** Average percentage of contact area when the load increases sharply.

Specimen Type	D1	D2	D3	L1	L2	L3
Average percentage of contact area (%)	92.6	91.2	91.3	92.6	91.4	91.8

**Table 3:** Material properties used in numerical models.

Property	Unit	CFRP	Aluminum (6061-T6)	Steel (42CrMo)
Elastic modulus, *E*_11_	MPa	161000 [26]	69,000	210,000
Elastic modulus, *E*_22_	MPa	8700 [27]	-	-
Elastic modulus, *E*_33_	MPa	8700 [27]	-	-
Poisson’s ratio, *ν*_12_	-	0.32 [27]	0.33	0.3
Poisson’s ratio, *ν*_13_	-	0.32 [27]	-	-
Poisson’s ratio, *ν*_23_	-	0.38 [27]	-	-
Shear modulus, G_12_	MPa	7800 [27]	-	-
Shear modulus, G_23_	MPa	3094 [27]	-	-
Shear modulus, G_13_	MPa	7800 [27]	-	-

## Data Availability

All data used to support the findings of this study are available from the corresponding author upon request.

## Nomenclature

A	Cross-sectional area of specimens
CFRP	Carbon-fiber-reinforced polymer
DIC	Digital image correlation
D	Diameter of specimens
FEM	Finite element model
*g_min_*	Required minimum gap
*g_max_*	Recommended maximum gap
*h*	Actual depth of groove in aluminum plate
*L*	Length of specimens
*P*	Contact force
*R*	Outer radius of aluminum plate
*r*	Inner radius of aluminum plate
*σ*	Longitudinal tensile stress
*μ*	Friction coefficient
